# Supplementary figures and images for: Transporter-Mediated Uptake of Microcystin-LR in Human Trophoblasts: Regulation By Oxygen Concentration and Cell Fusion
Source: bioRxiv. 2026 Mar 25:2026.03.22.713491. Preprint. [Version 1] doi: 10.64898/2026.03.22.713491 (PMC13041809; doi:10.64898/2026.03.22.713491)

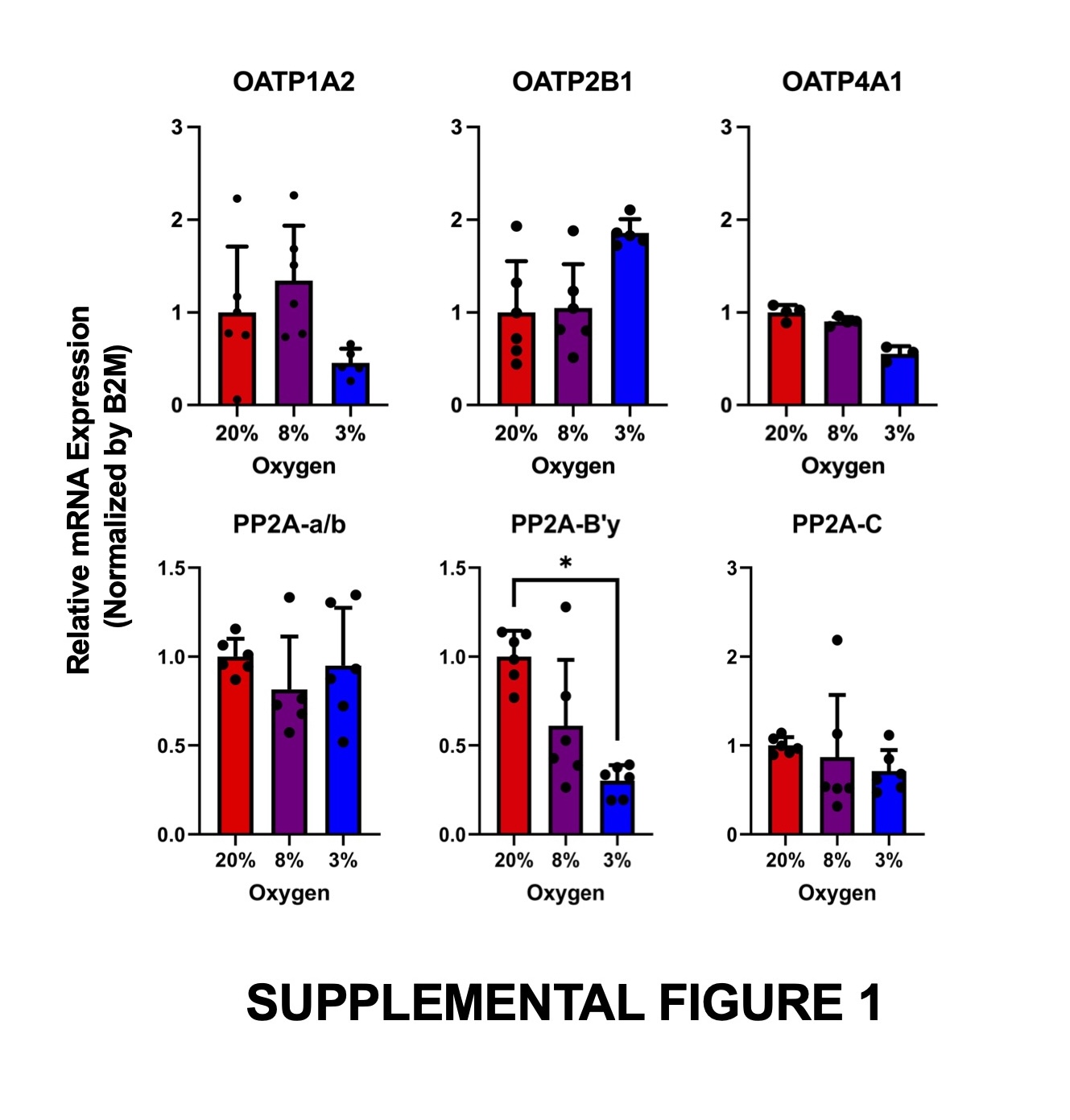

Supplement: Supplement 1 [file media-1.jpg]

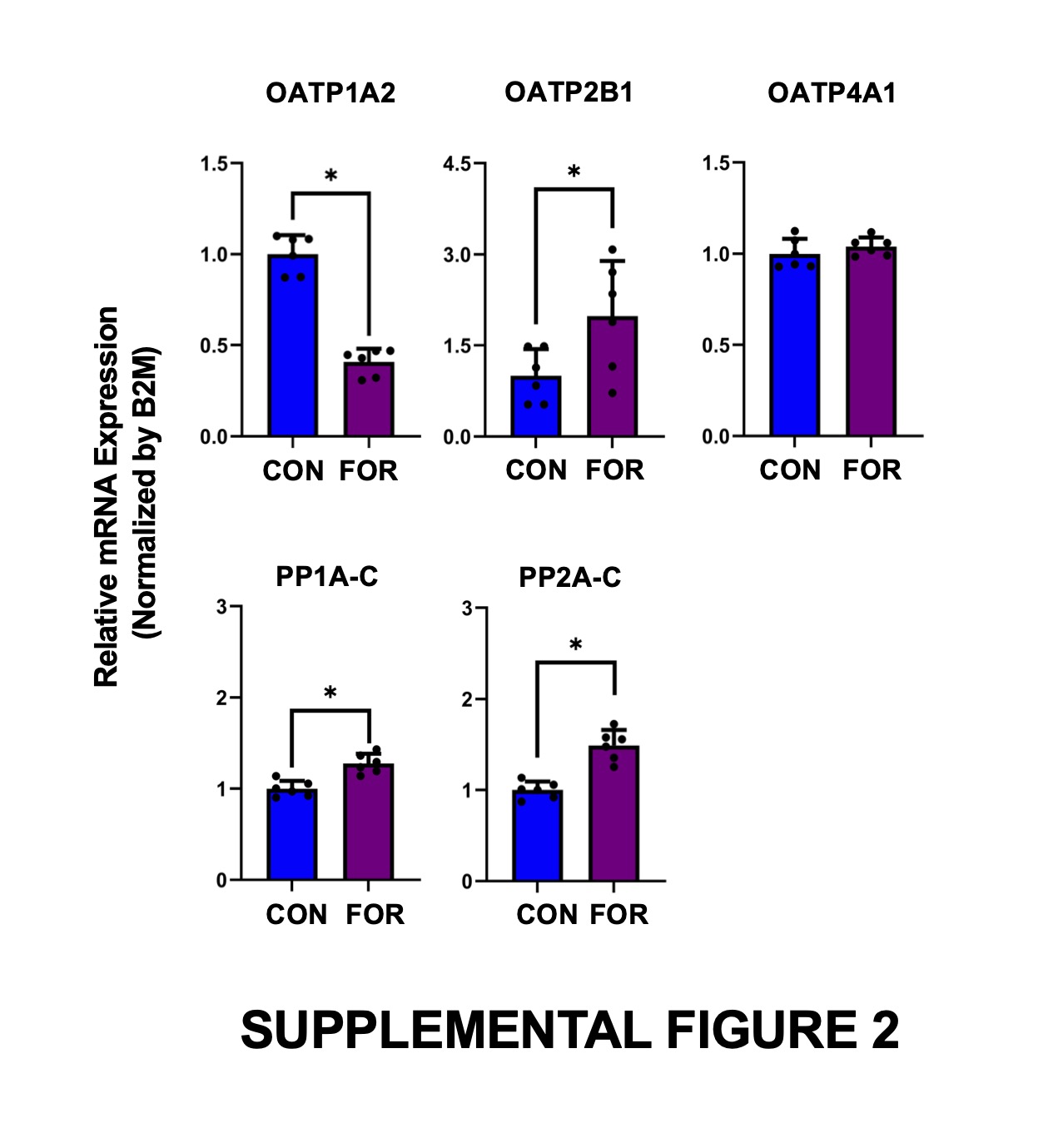

Supplement: Supplement 2 [file media-2.jpg]
